# Spermatogonial stem cells as a therapeutic alternative for fertility preservation of prepubertal boys

**DOI:** 10.1590/S1679-45082015RB3456

**Published:** 2015

**Authors:** Andrea Giannotti Galuppo

**Affiliations:** 1 Instituto de Ciências Básicas da Saúde, Universidade Federal do Rio Grande do Sul, Porto Alegre, RS, Brazil.

**Keywords:** Stem cells, Spermatogonia, Infertility, male, Cell culture techniques, Child

## Abstract

Spermatogonial stem cells, which exist in the testicles since birth, are progenitors cells of male gametes. These cells are critical for the process of spermatogenesis, and not able to produce mature sperm cells before puberty due to their dependency of hormonal stimuli. This characteristic of the reproductive system limits the preservation of fertility only to males who are able to produce an ejaculate. This fact puts some light on the increase in survival rates of childhood cancer over the past decades because of improvements in the diagnosis and effective treatment in pediatric cancer patients. Therefore, we highlight one of the most important challenges concerning male fertility preservation that is the toxic effect of cancer therapy on reproductive function, especially the spermatogenesis. Currently, the experimental alternative for fertility preservation of prepubertal boys is the testicular tissue cryopreservationfor, for future isolation and spermatogonial stem cells transplantation, in order to restore the spermatogenesis. We present a brief review on isolation, characterization and culture conditions for the *in vitro* proliferation of spermatogonial stem cells, as well as the future perspectives as an alternative for fertility preservation in prepubertal boys. The possibility of restoring male fertility constitutes a research tool with an huge potential in basic and applied science. The development of these techniques may be a hope for the future of fertility preservation in cases that no other options exist, *e.g*, pediatric cancer patients.

## INTRODUCTION

Spermatogonial stem cells (SSC), which are already present at birth, are stem cells of the testicle. They are the progenitors of male gametes and critical for the process of spermatogenesis.^(1,2)^ These cells are not able to produce mature sperm cells before puberty, which will occur after hormonal stimuli. This biological characteristic of the reproductive system limits the fertility preservation only to males who are able to produce an ejaculate, because the standard procedure for fertility preservation is semen cryopreservation.^(3-5)^

One of the most important challenges concerning male fertility preservation is the toxic effect of cancer therapy on reproductive function, especially spermatogenesis, which leads to infertility.^(2,6)^ Data related to toxic effects of cancer therapy in young men showed that 1 in every 5,000 is at risk for infertility.^(4)^ Over the past decades the survival rates for childhood cancer have increased due to improvements in the diagnosis and effective treatment.^(4)^ Along with this success has come the need to reduce treatment-related sequelae and optimize the quality of life of children undergoing treatment for cancer.^(7)^

Currently, the experimental alternative studied to restore spermatogenesis of postchemotherapy in prepubertal boys is the testicular tissue cryopreservation for future SSC retrieval and transplantation. Because restore of spermatogenesis in animal models was proved to be possible after SSC transplantation,^(8,9)^ it is important to continue with the efforts to successfully restore the fertility in men. Developing a technique to expand SSC for autologous transplantation or to differentiate SSC into mature sperm would be of great value.^(10)^ As a result, the isolation, storage and transplantation of SSC may become available for prepubertal boys diagnosed with cancer.^(3)^ We present a brief review on isolation, characterization and culture conditions for the *in vitro* proliferation of SSC, as well as the future perspectives as an alternative for fertility preservation in prepubertal boys.

## SPERMATOGONIAL STEM CELLS ISOLATION

Previous studies on isolation of testicular cells were based on these cells potential to self-renewal and their ability to transmit genes from one generation to another.^(11,12)^ These studies described the isolation of cells followed by transplantation into an infertile receptor, with no *in vitro* culture or characterization of the isolated cells.^(12,13)^ However, considering the success in spermatogenesis establishment of infertile receptors, the development of protocols for SSC isolation and purification comprise an important tool to allow the study of cellular biology of these cells and improve the SSC transplant protocols.^(14)^ The first step is the optimization of isolation procedures to improve purity of SSC, avoiding contamination with other cell types. This is possible through identification and characterization of the putative spermatogonia stem cell, since different populations of spermatogonia exist.^(15)^ The SSC are commonly isolated by enzymatic digestion, which usually includes a combination of enzymes like collagenases, trypsin, and DNAse.^(16)^ The cells obtained have to be submitted to a purification method. To get the most pure SSC population, different methods were developed, such as the morphology-based selection associated with differential plating, extracellular matrix selection, fluorescent-activated cell sorting (FACS) and magnetic-activated cell sorting (MACS).

The morphology-based selection is the simplest and cost effectively method, however, it has the lowest efficiency.^(17)^ Because it is based on the enzymatic isolation of the cells followed by its plating in different times, it produces highly contaminated samples with different types of testicular cells, as Sertoli cells, Leydig cells, myoid cells, and fibroblasts.^(17,18)^ These cells can release growth factors, hormones and extracellular matrix and may interfere in the SSC *in vitro* self-renewal and proliferation.^(18)^ The extracellular matrix based selection uses different extracellular proteins, such as laminin and fibronectin, to promote cell adhesion to SSC. These subtracts have the potential to bind other extracellular matrix components and they are widely used in *in vitro* cells culture to help cell attachment and promote cell proliferation.^(19)^ As the SSC have weak potential for adhesion, the promotion of their attachment by coating of substrates is necessary to its *in vitro* maintenance*.* ^(19)^ The high pure SSC population can be obtained by the use of sorting assays like FACS and MACS. Both methods can be used which several markers that may enhance the selection of SSC. Although these techniques can provide a highly pure cell population, they have shortcomings in resource-consuming procedures and technical complexity, which result in low cell yield and viability.^(16,19)^

The combination of isolation techniques has been used as an attempt to obtain highly pure cell suspensions. The majority of protocols begin with a differential plating of the initial cell suspension to eliminate other testicular cell types. This technique separates cells according to their distinct attachment capabilities. Cells are plated directly into tissue culture dishes or matrix-coated dishes using gelatin, laminin, fibronectin or collagen. After a period, non-attached cells, that include SSC, are collected and submitted to sorting-based strategies.^(19,20)^ A recent study showed effective elimination of contaminated cells and highly pure harvest of SSCs by the use of two-step purification. First, the collected SSCs were cultured on somatic and Sertoli cells and after detachment, cells were purified by centrifugation in a bovine serum albumin (BSA) density gradient. This method was able to remove most of the non-SSC and the purity of cell suspension reached more than 91.5%.^(19)^

## SPERMATOGONIAL STEM CELLS CELLULAR MARKERS

Considering that the main goal of SSC isolation, culture and transplantation is to preserve and restore the fertility, and also that majority of the patients who will benefit from SSC transplantation have cancer when testicle biopsies are collected, the inherent risk of reintroduce contaminated malignant cells into patients must be eliminated. Although several markers were identified in SSC, and helped the cell isolation process, none of them proved to be specific for this population. The first panel on markers used for SSC isolation by FACS was based on positive identification for β1-integrin (CD29) and a6-integrin (CD49f), and negative for av-integrin (CD51).^(21)^ However, after that initial study, other SSC markers were identified: Thy1 (CD90), the CD9, the a1 receptor from glial cell line-derived neurotrophic factor (GFRα1), SSEA-4 and the E-cadherin.^(10,20,22)^ Some negative markers were also used, such as the c-Kit (CD117) and CD45.^(22)^ The achievement of highly purified SSC populations with the combination of positive and negative selection markers has proven to be essential to enrich undifferentiated spermatogonia and remove the malignant contamination.^(1)^ Despite, it was not possible yet to identify a combination of positive and negative markers to provide a pure SSC suspension.^(23)^ A recent study demonstrated that the combination of CD90 (positive marker) and CD45 (negative marker) resulted in cancer-free germ cells suspension.^(10)^ However, post-sort purity checks are required to confirm removal of cells with potential cancer phenotype.^(10)^

## *IN VITRO* CULTURE CONDITIONS OF SPERMATOGONIAL STEM CELLS

Spermatogonial stem cells are present in reduced numbers in the seminiferous tubules, around 0.03% of these cells in testicles. SSC needs to be propagated to increase the number of undifferentiated cells required for transplantation.^(10,24)^ To achieve this increase an *in vitro* environment should be provided, as similar as possible to the SSC niche in the testicles. The SSC niche is composed by different supporting cells, like Sertoli, peritubular, myoid and Leydig cells.^(15)^ However, the enzymatic treatment for tissue dissociation and cell isolation destroy the compartments of the microenvironment, *i.e.*, interstitial, basal, intraepithelial, and adluminal microenvironment.^(16)^

The basic culture system developed for SSC relies on a culture medium supplemented with hormones, growth factors and a feeder layer of somatic cells like mouse embryonic fibroblast (MEF), *i.e*., the STO cell line or Sertoli cells.^(25)^ A key factor for the majority of cell cultures, the serum, is detrimental to the SSC proliferation.^(26)^ After the evaluation of different serum concentrations for SSC culture, none serum concentration was able to enhance proliferation compared with serum-free condition.^(26)^ The use of Knockout™ Serum Replacement (KSR) and BSA as an alternative to serum allowed SSC proliferation for facilitating cell cycle progression.^(27)^ To fill the lack of hormones or growth factors provided by the serum, it is required the addition of purified proteins and supplements.^(27)^ Some of the identified growth factors required for SSC proliferation are the glial cell line-derived neurotrophic factor (GDNF), GDNF family receptor a1 (GFRA1) and fibroblast growth factor 2 (FGF2).^(14,26)^

The use of a feeder layer of MEF or STO cells has a positive effect on the SSC maintenance because these cells release growth factors and cytokines that promote the conditioning of the media.^(9,28)^ Similarly MEF feeder layer supplies a friendly surface for SSC attachment. Similar to any other cell type, SSC are directly influenced by the topography, roughness and stiffness of substrates.^(28,29)^ All improvements in SSC culture conditions allowed long-term expansion of cells from different mouse strains and age groups. SSC were maintained undifferentiated for 6 months without loss of function and capability of reconstitute normal spermatogenesis after transplantation.^(26)^ However the clinical application of SSC requires the development of xeno-and feeder-free culture systems to avoid pathogen contaminations.^(30)^

## FUTURE POSSIBILITIES OF THE USE OF SPERMATOGONIAL STEM CELLS

Since the late 1990s spermatogenesis recovery in animal models is feasible. Since then a large progress has been made on the knowledge of the biology and characteristics of spermatogonial stem cells, as well as on the maintenance and cellular markers of the cells and on the identification of cellular markers of spermatogonial stem cells. The capability of modulate the *in vitro* conditions involved in the control of the self renewal versus differentiation decision of spermatogonial stem cells will lead to produce functional gametes *in vitro.* ^(26)^ This production associated with the transplantation techniques from animal models to humans allow the study of the molecular and cellular biology of male germ cell differentiation, and enable the development of new therapeutic strategies for infertility.^(15,26)^

The possibility of restoring fertility in men has a great potential in basic and applied science.^(14)^ The development of culture techniques may provide future hope for fertility preservation in cases without other options, *e.g*., in prepubertal cancer patients. Many health clinics are already cryopreserving testicular tissue from male cancer patients; but techniques should be developed to eliminate the risk of re-introducing malignant cells during the spermatogonial stem cells transplantation.^(1)^

Besides, a variety of studies showed that spermatogonial stem cells are able to differentiate into various cell types *in vitro*, such as cardiomyocytes and neural cells. It is important to note that these cells have several advantages compared with embryonic stem cells, including no ethical concerns about their use and origin, and present lower frequency of tumorigenesis and immune rejection.^(19)^ Based on these assumptions, spermatogonial stem cells might be one of the most promising candidates for clinical applications in cell therapy studies.^(19)^
