# Supplementary material for: Spermatogonial stem cells as a therapeutic alternative for fertility preservation of prepubertal boys
Source: Einstein (Sao Paulo). 2015 Oct-Dec;13(4):637–9. doi: 10.1590/S1679-45082015RB3456 (PMC4878644; doi:10.1590/S1679-45082015RB3456)
Supplement: Translation

# Translation: Espermatogônias-tronco como uma alternativa para a preservação da fertilidade de meninos pré-púberes

**Authors:** Andrea Giannotti Galuppo

**Affiliations:** 1 Instituto de Ciências Básicas da Saúde, Universidade Federal do Rio Grande do Sul, Porto Alegre, RS, Brasil.

**Keywords:** Células-tronco, Espermatogônias, Infertilidade masculina, Técnicas de cultura de células, Criança

## Abstract

As espermatogônias-tronco, presentes nos testículos desde o nascimento, são as células progenitoras dos gametas masculinos, e, desse modo, críticas para o processo de espermatogênese. Antes da puberdade, essas células não são capazes de produzir espermatozoides maduros, o que só ocorrerá após o estímulo hormonal. Essa característica do sistema reprodutivo limita a possibilidade de preservação da fertilidade apenas para homens capazes de produzir um ejaculado. Tal fato coloca em evidência o aumento nas taxas de sobrevivência de crianças com câncer nas últimas décadas, devido principalmente à melhora no diagnóstico e ao tratamento dos pacientes pediátricos. Dessa forma, destaca-se um dos mais importantes desafios relativos à preservação da fertilidade masculina, que é o efeito tóxico das terapias anticâncer para o sistema reprodutivo, especialmente a espermatogênese. Tendo isso em vista, a alternativa experimental atualmente estudada para a preservação da fertilidade de pacientes pré-púberes é a criopreservação de tecido testicular para futuro isolamento e transplante de espermatogônias-tronco, a fim de restabelecer a espermatogênese. Apresentamos aqui uma breve revisão sobre isolamento, caracterização e condições de cultivo para a proliferação de espermatogônias-tronco, bem como as futuras perspectivas, como alternativa para preservação da fertilidade de meninos pré-púberes. A possibilidade de restabelecer a fertilidade masculina é uma ferramenta de pesquisa com potencial enorme de uso na pesquisa básica e aplicada. O desenvolvimento dessas técnicas pode fornecer uma esperança futura de preservação de fertilidade nos casos em que não há nenhuma outra opção, como para os pacientes pediátricos de câncer.

## INTRODUÇÃO

As espermatogônias-tronco (ET), presentes desde o nascimento, são as células-tronco testículares. São as células progenitoras dos gametas masculinos e primordiais para o processo de espermatogênese.^(1,2)^ Antes da puberdade, essas células não são capazes de produzir espermatozoides maduros, o que só é possível após o estímulo hormonal. Essa característica biológica do sistema reprodutor limita a preservação da fertilidade apenas aos homens que são capazes de produzir um ejaculado, já que o procedimento padrão para preservação da fertilidade é a criopreservação de sêmen.^(3-5)^

Um dos principais desafios em relação à preservação da fertilidade masculina é o efeito tóxico da terapia oncológica na função reprodutiva, especialmente na espermatogênese, que pode levar à infertilidade.^(2,6)^ Os dados relacionados aos efeitos tóxicos da terapia oncológica em homens jovens mostram que 1 em cada 5.000 homens estão sob risco de infertilidade.^(4)^ Ao longo das ultimas décadas, a taxa de sobrevida para câncer na infância tem aumentado devido a melhorias no diagnóstico e tratamento.^(4)^ Com esse sucesso, apresenta-se a necessidade de reduzir sequelas e de melhorar a qualidade de vida de crianças submetidas ao tratamento para câncer.^(7)^

Atualmente, a alternativa experimental estudada para restaurar a espermatogênese após quimioterapia em meninos pré-púberes é a criopreservação do tecido testicular para futuro isolamento e transplante das ET. Já foi provado, em modelos animais, que é possível restaurar a espermatogênese após o transplante dessas células.^(8,9)^ Dessa forma, é importante continuar os esforços para restaurar com sucesso a fertilidade masculina. O desenvolvimento de protocolos visando à proliferação das ET *in vitro* para transplante autólogo ou para diferenciação em espermatozoides maduros seria de grande valor.^(10)^Como resultado, o isolamento, o armazenamento e o transplante de ET podem se tornar disponíveis para meninos pré-púberes diagnosticados com câncer.^(3)^ Apresentamos aqui uma breve revisão sobre o isolamento, caracterização e condições de cultura para proliferação de ET *in vitro*, bem como as perspectivas futuras, como uma alternativa após tratamento para restauração da fertilidade.

## ISOLAMENTO DE ESPERMATOGÔNIAS-TRONCO

Estudos recentes de isolamento de células testiculares foram baseados em seu potencial de autorrenovação e habilidade de transmitir genes de uma geração para outra.^(11,12)^ Tais estudos descreveram isolamento de células, seguido por transplante para um receptor infértil, sem cultura *in vitro* ou caracterização de células isoladas.^(12,13)^ No entanto, considerando o sucesso no estabelecimento da espermatogênese de receptores inférteis, o desenvolvimento de protocolos para isolamento e purificação de ET constitui uma ferramenta importante para permitir o estudo da biologia celular dessas células e melhora dos protocolos de transplante de ET.^(14)^ O primeiro passo é o aperfeiçoamento dos procedimentos de isolamento para melhorar a pureza das ET, evitando, assim, contaminação com outros tipos celulares. Isso é possível por meio da identificação e caracterização de ET, já que há populações diferentes de espermatogônias.^(15)^ As ET são comumente isoladas por digestão enzimática, geralmente uma combinação de enzimas como colagenases, tripsina e DNAse.^(16)^ As células obtidas devem ser submetidas a métodos de purificação. Para conseguir uma população de ET mais pura, métodos diferentes foram desenvolvidos, como seleção baseada em morfologia associada com cultura diferencial, seleção de matriz celular, *fluorescent*-*activated cell sorting* (FACS) e *magnetic-activated cell sorting* (MACS).

A seleção com base na morfologia é o método mais simples e com menor custo, porém é também o menos eficiente.^(17)^ É baseado no tratamento enzimático do tecido para obtenção das células, seguido do plaqueamento das células em tempos diferentes; produz amostras altamente contaminadas com diferentes tipos de células testiculares, como células de Sertoli, células de Leydig, células mioides e fibroblastos.^(17,18)^ Essas células podem liberar fatores de crescimento, hormônios e matriz extracelular, e interferir na autorrenovação e proliferação de ET *in vitro*.^(18)^ A seleção com auxílio de proteínas da matriz extracelular se baseia no uso de proteínas extracelulares, como laminina e fibronectina, para facilitar a adesão das ET ao substrato de cultura. Esses substratos têm potencial de ligar outros componentes da matriz extracelular e são muito utilizados na cultura *in vitro* de células, para auxiliar a adesão celular e promover sua proliferação.^(19)^ Devido às ET possuírem baixo potencial de adesão, o uso de proteínas de adesão, para facilitar a adesão das ET ao substrato, é necessário para sua manutenção *in vitro.* ^(19)^Uma população altamente pura de ET pode ser obtida por meio do uso de técnicas de separação, como o FACS e MACS. Ambos os métodos podem ser utilizados com diversos marcadores, melhorando a seleção de ET. Apesar de essas técnicas possibilitarem a obtenção de uma população altamente pura de células, trata-se de procedimentos de alto custo, extremamente complexos e que podem promover danos às células, o que resulta em baixa proliferação e viabilidade celular.^(16,19)^

A combinação de técnicas de isolamento tem sido utilizada como uma tentativa para obter suspensões celulares altamente puras. A maioria dos protocolos começa com uma suspenção celular inicial, para eliminar outros tipos celulares dos testículos. Essa técnica separa as células pela diferença de capacidade de adesão ao substrato. As células são cultivadas diretamente em placa para cultura de células, com gelatina, laminina, fibronectina ou colágeno. Após um período de tempo, as células não aderidas ao substrato, que incluem as ET, são coletadas e submetidas às técnicas de isolamento.^(19,20)^ Estudo recente mostrou, por meio do uso da purificação em duas etapas, a eliminação efetiva de células contaminadas e a coleta de ET altamente puras. Inicialmente, as ET coletadas foram cultivadas células somáticas e de Sertoli, e, depois da separação, as células foram purificadas por centrifugação em gradiente de densidade com albumina do soro bovino (BSA). Esse método foi capaz de remover a maioria das células não TE e a purificação da suspensão celular alcançou mais de 91,5%.^(19)^

## MARCADORES CELULARES DE ESPERMATOGÔNIAS-TRONCO

Considerando que o principal objetivo do isolamento, cultura e transplante de ET é a preservação e a restauração da fertilidade, e que a maioria dos pacientes que se beneficiarão a partir do transplante de ET tem câncer quando as biópsias dos testículos são coletadas, o risco inerente de reintroduzir células malignas nos pacientes deve ser eliminado. Apesar da identificação de diversos marcadores em ET e da ajuda no processo de isolamento celular, nenhum deles provou ser especifico para essa população. O primeiro painel de marcadores utilizados para isolamento das ET por meio de FACS foi baseado na identificação positiva por β1-integrina (CD29) e a6-integrina (CD49f), e negativa para av-integrina (CD51).^(21)^ Porém, após estudo inicial, outros marcadores de ET foram identificados: Thy1 (CD90), o CD9, o receptor a1 derivado do fator neurotrófico das células da glia (GFRα1), SSEA-4 e a E-caderina.^(10,20,22)^ Alguns marcadores negativos também foram utilizados, tais como c-Kit (CD117) e CD45.^(22)^ Isolar uma população de ET altamente purificada por meio da combinação de marcadores de seleção positivos e negativos é essencial para obter espermatogônias indiferenciadas e remover a contaminação maligna.^(1)^Porém, ainda não foi possível identificar uma combinação de marcadores positivos e negativos que produza uma suspensão de ET pura.^(23)^ Um estudo recente demonstrou que a combinação de CD90 (marcador positivo) e CD45 (marcador negativo) resultou em uma suspensão de células germinativas livres de células cancerígenas.^(10)^ Porém, a verificação da pureza da amostra pós-seleção é necessária para confirmar a remoção de células com potencial fenótipo cancerígeno.^(10)^

## CONDIÇÕES DE CULTURA *IN VITRO* DE ESPERMATOGÔNIAS-TRONCO

Espermatogônias-tronco estão presentes em número reduzido nos túbulos seminíferos, aproximadamente 0,03% dessas células nos testículos. As ET precisam ser proliferadas para aumentarem o número de células indiferenciadas necessárias para o transplante.^(10,24)^ Para alcançar essa proliferação, um ambiente *in vitro* o mais similar possível ao nicho das ET nos testículos deve ser disponibilizado. O nicho das ET é composto por diferentes tipos celulares, como as células de Sertoli, peritubulares, mioides e de Leydig.^(15)^No entanto, o tratamento enzimático para dissociação do tecido e isolamento das ET destrói os compartimentos do microambiente onde ela se encontra *in vivo*, como, por exemplo, os ambientes intersticial, basal, intraepitelial e microambiente adluminal.^(16)^

O sistema de cultura básico desenvolvido para as ET depende do uso de um meio de cultura suplementado com hormônios, fatores de crescimento e uma camada alimentadora de células somáticas, como fibroblastos embrionários de camundongos (MEF - *mouse embryonic fibroblasts*), linhagem de células STO ou Sertoli.^(25)^Um fator fundamental para maioria das culturas celulares, o soro fetal, provou ser prejudicial para proliferação das ET.^(26)^ Após avaliação do uso de diferentes concentrações de soro fetal para cultura de ET, nenhuma foi capaz de melhorar a proliferação comparada a condição livre de soro.^(26)^ O uso de Knockout™ Serum Replacement (KSR) e BSA como uma alternativa ao soro possibilitou a proliferação das ET por meio da facilitação de progressão do ciclo celular.^(27)^ Para preencher a falta de hormônios ou fatores de crescimento presentes no soro, é necessário adicionar proteínas purificadas e suplementos.^(27)^Alguns dos fatores de crescimento identificados que são necessários para proliferação das ET são o fator neurotrófico derivado de células da glia (GDNF), o receptor a1 derivado do GDNF (GFRA1) e o fator crescimento de fibroblasto 2 (FGF2).^(14,26)^

O uso de camada alimentadora com MEF ou STO tem um efeito positivo na manutenção das ET, pois as células da camada alimentadora liberam fatores de crescimento e citocinas, que promovem o condicionamento do meio de cultura.^(9,28)^ De modo similar, a camada alimentadora com MEF provê uma superfície mais adequada para a adesão das ET. Como qualquer outro tipo de célula, as ET são diretamente influenciadas pela topografia, robustez e firmeza dos substratos.^(28,29)^ Todas as melhorias nas condições de cultura da ET permitiram a manutenção a longo prazo de linhagens obtidas de diferentes camundongos e faixas etárias. As ET foram mantidas indiferenciadas por 6 meses sem perda de função e capacidade de retomar a espermatogenese normal após o transplante.^(26)^ Porém, a aplicação clínica de ET requer o desenvolvimento de sistemas de cultivo livre do uso de produtos de origem animal para evitar possíveis contaminações patogênicas.^(30)^

## POSSIBILIDADES FUTURAS DO USO DAS ESPERMATOGÔNIAS TRONCO

Desde 1990, o restabelecimento da espermatogênese em modelos animais foi comprovado. A partir daí, um grande progresso aconteceu em relação ao conhecimento da biologia e das características das espermatogônias-tronco, assim como no conhecimento sobre a manutenção dessas células *in vitro* e na identificação de marcadores celulares de espermatogônias-tronco. A capacidade de modular as condições *in vitro* envolvidas no controle entre autorrenovação *versus* decisões de diferenciação das espermatogônias-tronco levar à produção de gametas funcionais *in vitro.* ^(26)^ Essa produção, associada às técnicas de transplante de modelos animais para humanos, permitem o estudo da biologia molecular e celular da diferenciação de células germinativas masculinas, e possibilita o desenvolvimento de novas estratégias terapêuticas para tratamento da infertilidade.^(15,26)^

A possibilidade de restaurar a fertilidade em homens tem enorme potencial em pesquisa básica e ciência aplicada.^(14)^ O desenvolvimento de técnicas de cultura pode oferecer esperança para a futura preservação da fertilidade em casos em que não há outras opções, como de pacientes com câncer na pré-puberdade. Muitas clínicas já promovem a criopreservação de tecido testicular de homens com câncer, porém é importante desenvolver técnicas para eliminar o risco de reintroduzir células malignas durante o transplante de espermatogônias-tronco.^(1)^

Além disso, diversos estudos também mostram que espermatogônias-tronco são capazes de se diferenciar em vários tipos de células *in vitro*, tais como cardiomiócitos e células neurais. É importante destacar que essas células têm diversas vantagens em comparação as células-tronco embrionárias, incluindo a ausência de problemas éticos associados a sua origem, e também a baixa frequência de tumorigênese e rejeição imune.^(19)^ Considerando todas as questões, as espermatogônias-tronco podem ser uma opção promissora para aplicação clinica em estudos de terapia celular.^(19)^
